# Development of innovative methodology for determination of 6-thioguanine in whole blood erythrocytes by HPLC–PDA-based technique for medical diagnostics purposes

**DOI:** 10.1038/s41598-023-41426-5

**Published:** 2023-08-29

**Authors:** Maciej Noga, Marcin Zakrzewski, Dorota Wianowska, Maciej Gnatowski, Łukasz Paprotny, Kamil Jurowski

**Affiliations:** 1 Department of Regulatory and Forensic Toxicology, Institute of Medical Expertises, Ul. Aleksandrowska 67/93, 91-205 Lodz, Poland; 2 ALAB Laboratories, Research and Development Centre, Ul. Ceramiczna 1, 20-150 Lublin, Poland; 3 Department of Chromatographic Methods, Faculty of Chemistry, Maria Curie-Skłodowska University, Pl. Maria Curie-Skłodowska 3, 20-031 Lublin, Poland; 4 Institute of Medical Studies, MedicalCollege, Rzeszów University, Al. mjrr. W. Kopisto 2a, 35-959 Rzeszow, Poland

**Keywords:** Analytical chemistry, Analytical biochemistry, Medical research, Diagnostic markers

## Abstract

6-Thioguanine is an immunosuppressive drug, an analogue of guanine, applied to treat acute leukemia and inflammatory bowel disease. Excessive use of 6-thioguanine during clinical treatment may cause side effects. Moreover, providing a dose too low will be ineffective. Therefore, there is a critical need for a rapid, selective and routine approach to quantifying 6-thioguanine in body fluids to support a clinical application. A fully validated HPLC method has been developed to determine 6-thioguanine in whole blood samples using 5-bromouracil as an internal standard. 6-Thioguanine nucleotides were released from erythrocytes by perchloric acid, and then hydrolysed at 100 °C to the parent thiopurine, 6-thioguanine. The following validation parameters of the method were determined: specificity/selectivity, linearity range (479–17,118 ng/mL, R > 0.992), limits of detection (150 ng/mL) and quantification (479 ng/mL), accuracy (− 5.6 < Bias < 14.7), repeatability (CV 1.30–3.24%), intermediate precision (CV 4.19–5.78%), extraction recovery (79.1–103.6%) and carryover. Furthermore, the stability of the drug in whole blood samples under various storage conditions was investigated. The suggested method is suitable for determining 6-thioguanine in whole blood erythrocyte samples for drug level monitoring, thus correct dosing.

## Introduction

6-Thioguanine (6-TG) is a purine analogue of guanine; more precisely, it is a sulphur derivative of guanine^1^. 6-TG belonged to a thiopurine family and was introduced in 1950 as a treatment for leukemia. Fifty years later, clinicians started using it to treat inflammatory bowel disease, those who have not received azathioprine (AZA) or mercaptopurine (MP) due to dosage-limiting adverse events or suboptimal response^2, 3^. However, concerns about the hepatotoxicity profile have been hampered by widespread use, specifically nodular regenerative hyperplasia (NRH)^4^. 6-TG is also used in the clinical treatment of acquired immunodeficiency syndrome, autoimmune diseases such as psoriasis and chemotherapy for acute leukaemias, particularly acute myeloid leukaemia and acute lymphoblastic leukemia^5, 6^. The additional risk associated with 6-TG may be the development of lymphoma and skin cancer, as demonstrated in the case of AZA/MP and anti-TNF therapies^7, 8^. Therefore, it is important to regularly verify the level of 6-TG to confirm the correct dose of the drug or, in case of abnormal results, to adjust the dose. Thiopurines, including 6-TG, have an immunosuppressive effect by incorporating their pharmacologically active metabolites, 6-thioguanine nucleotides (6-TGN), into nucleic acids^9^. Consequently, this leads to inhibition of lymphocyte proliferation, including their significant role in inducing apoptosis^10^. Although the mechanisms causing leukemic cell resistance to 6-thioguanine are currently misunderstood^11^, it has been suggested that reduction or absence of hypoxanthine–guanine phosphoribosyl transferase (HGPT) activity together with altered thiopurine S-methyltransferase (TPMT) activity stimulate sensitivity and resistance to this compounds^12^. Furthermore, defects in the DNA mismatch repair system have been shown to stimulate acquired resistance to numerous anticancer pharmaceutical compounds, including 6-thioguanine^13^. Measurement of 6-TG erythrocyte metabolite levels helps determine the adequacy of AZA dosage and can be used to optimise the dose of antimetabolite therapy dose to achieve an improved clinical response without inducing leucopenia^14^. Combining infliximab and thiopurines (such as 6-thioguanine) in patients with inflammatory bowel diseases is more effective than monotherapy. Measurement of TPMT activity is encouraged before starting treatment of patients with thiopurine drugs such as 6-thioguanine, allowing adjustment of thiopurine dose or complete avoidance of the drug entirely^15^. In many ethnicities, TPMT polymorphisms result in decreased (10% prevalence) or absent (0.3%) TPMT activity^16^. Individuals who are homozygous or heterozygous for these types of genetic variation may have increased levels of TGN metabolites, increasing the risk of drug-induced bone marrow toxicity due to accumulation of the unmetabolised drug^17^. 6-TGN levels greater than 230 pmol/8 × 10^8^ RBC have been associated with improved outcomes in patients on monotherapy. A 6-thioguanine level of 125 pmol/8 × 10^8^ RBCs or greater may be adequate to achieve therapeutic infliximab levels^18^. Generally, 6-TGN levels greater than 235 pmol/8 × 10^8^ RBC are recommended as a therapeutic cut-off level for patients on AZA or 6-MP^19^. A safe and therapeutic level is between 500 and 800 pmol/8 × 10^8^ RBCs^20^. For 6-TG therapy in patients with IBD, no precise therapeutic or toxic 6-TGN levels have been described yet. However, hepatotoxicity, particularly nodular regenerative hyperplasia (NRH) and venoocclusive disease (VOD), is dose-dependent and related to 6-TGN levels higher than 1000 pmol/8 × 10^8^ RBCs^21^. Overuse of 6-TG during clinical treatment may have extra-toxic side effects^6^. Furthermore, underdosing will be ineffective. Therefore, a quick and fully validated approach to quantitatively determining 6-TG in body fluids is critical to aid in clinical use. Currently, immunoassays and high-performance liquid chromatography (HPLC) are widespread, practical, and sensitive techniques in therapeutic drug monitoring^22, 23^.

Until now, several analytical approaches have been described for determining thiopurine metabolites applying UPLC-MS/MS^24^ and LC–MS/MS^25–27^. The defined methods vary in biological matrix, sample preparation procedure, and chemical form of the analytes. However, none of the 6-TGs allowed above can be quantified directly from red blood cells by HPLC alone. Numerous laboratories worldwide observe the metabolites of thiopurine to personalise the treatment. 6-TG is currently measured in red blood cells^11^ and whole blood^28^. Leukocyte 6-TG levels ranged from 100 to 2305 pmol/mg DNA, while erythrocyte 6-TG levels ranged from 64 to 1038 pmol/8 × 10(8) red blood cells; leukocyte DNA 6T-G levels correlated directly with erythrocyte 6-TG levels^29^. Most of the effects of thiopurine, such as DNA incorporation and immunosuppression, occur in white blood cells rather than red blood cells. However, these cells exist abundantly and are effortlessly separated, making them a rational alternative matrix^30^.

The purpose of our work was to develop a fully validated HPLC method for the rapid and selective quantitative determination of 6-TG in whole blood erythrocytes for diagnostic purposes. The method has been rigorously validated. Furthermore, specific validation parameters and clinical evaluation have been determined and demonstrated that the method suits the routine determination of 6-TG in patients. Our goal was to develop a routine method in the first place so that any laboratory could repeat the entire procedure for clinical studies. To our knowledge, this article is the only simple, inexpensive, sensitive, well-characterised, robust, and fully validated HPLC method for monitoring 6-TG in whole blood.

## Results and discussion

The HPLC method was developed for the determination of 6-thioguanine (6-TG) in whole blood samples containing red blood cells (RBCs) using 5-bromouracil (5-BU) as internal standard (IS) was developed. A mobile phase consisting of ACN/buffer KH_2_PO_4_ (3/97, v/v) with 50 µl H_3_PO_4_ in isocratic flow was used. 6-TG was detected at wavelength 341 nm and 6-BU at 280 nm. Under the chromatographic conditions, both the compounds and the biological background were well separated. A simple and rapid method of releasing 6-thioguanine nucleotides from erythrocytes by perchloric acid and then hydrolysed (100 °C) to the parent thiopurine (6-TG) was used to prepare the samples and then hydrolysed (100 °C) to the parent thiopurine (6-TG). By converting the released 6-TG content to 6-TGN, the amount of 6-TGN present in the erythrocytes can be determined. The method has been fully validated according to EMA_22_, ICH_23_ and ISO 17025_24_. Such analytical parameters are described as specificity, selectivity, linearity range, extraction recovery, and repeatability, precision (intraday and intra-day precision), accuracy, LOD, and LOQ.

### Specificity

**Figure 1:**
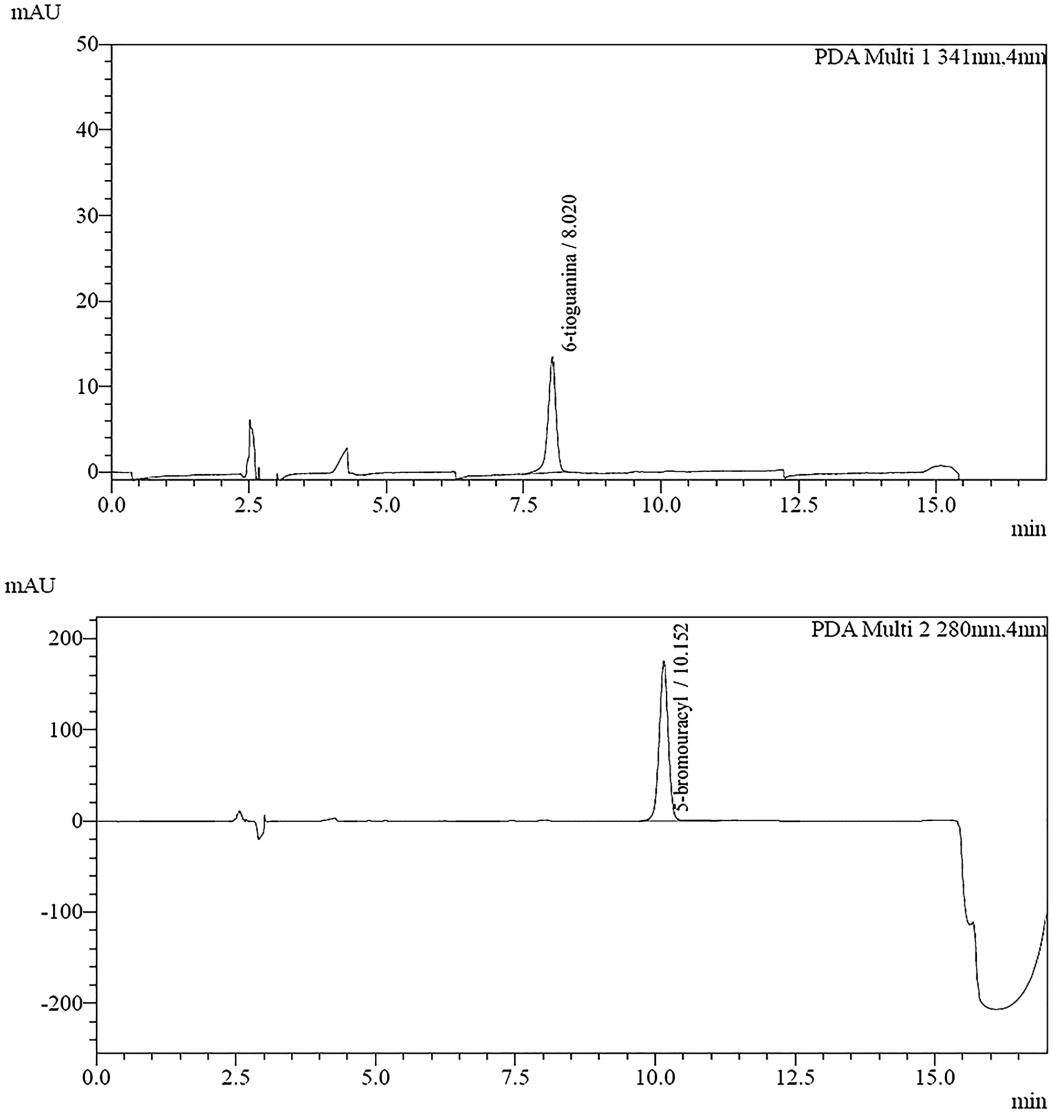
Chromatograms of an aqueous solution of 6-TG and IS.

**Figure 2:**
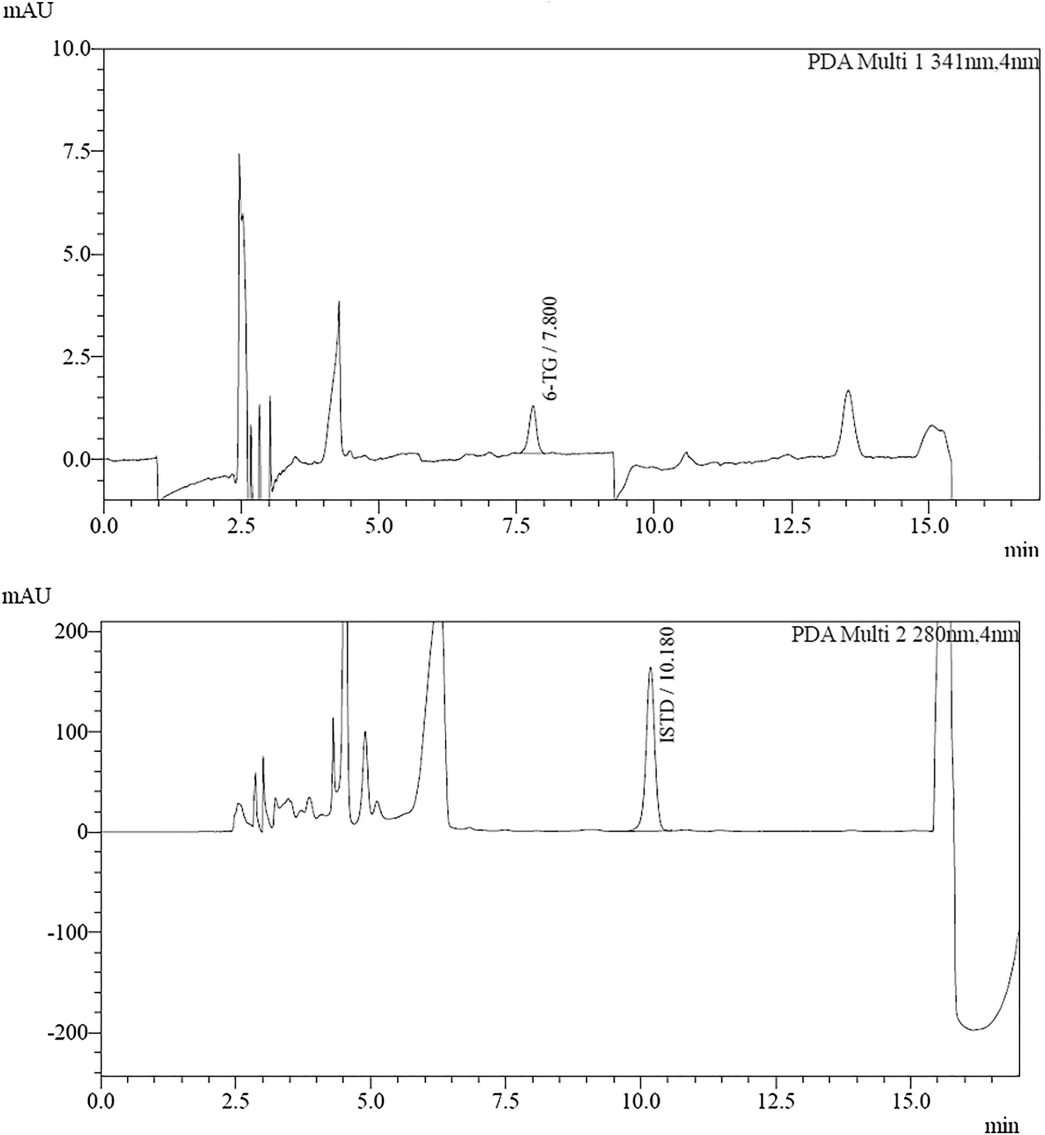
Chromatograms of a sample devoid of 6-TG and IS.

The method turned out to be specific in the assumed experimental conditions. A comparison of the chromatogram of the sample of the aqueous solution of the 6-thioguanine standard and the internal standard (Fig. 1) shows the lack of the peaks of substances that are eluting, respectively, during the retention of the internal standard and the retention of the analyte standards. Similarly, a comparison of the chromatogram showing the separation of the sample without analytes and the IS (Fig. 2) reveals the absence of substance peaks that could negatively affect the result of the determination of the test substance.

**Figure 3:**
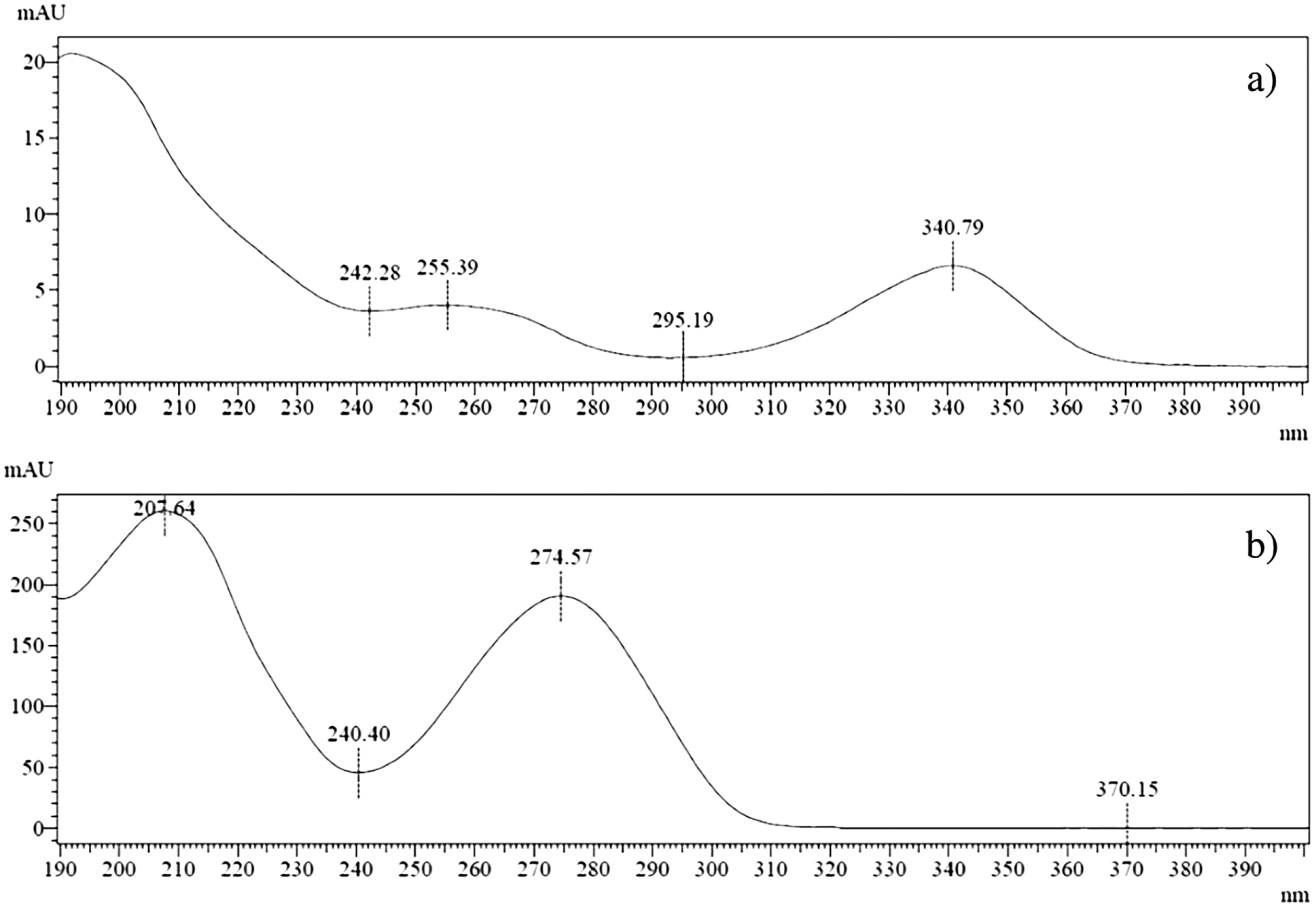
Standard spectrums: (**a**) 6-thioguanine in the test sample at 2000 ng/ml, (**b**) internal standard in serum.

**Figure 4:**
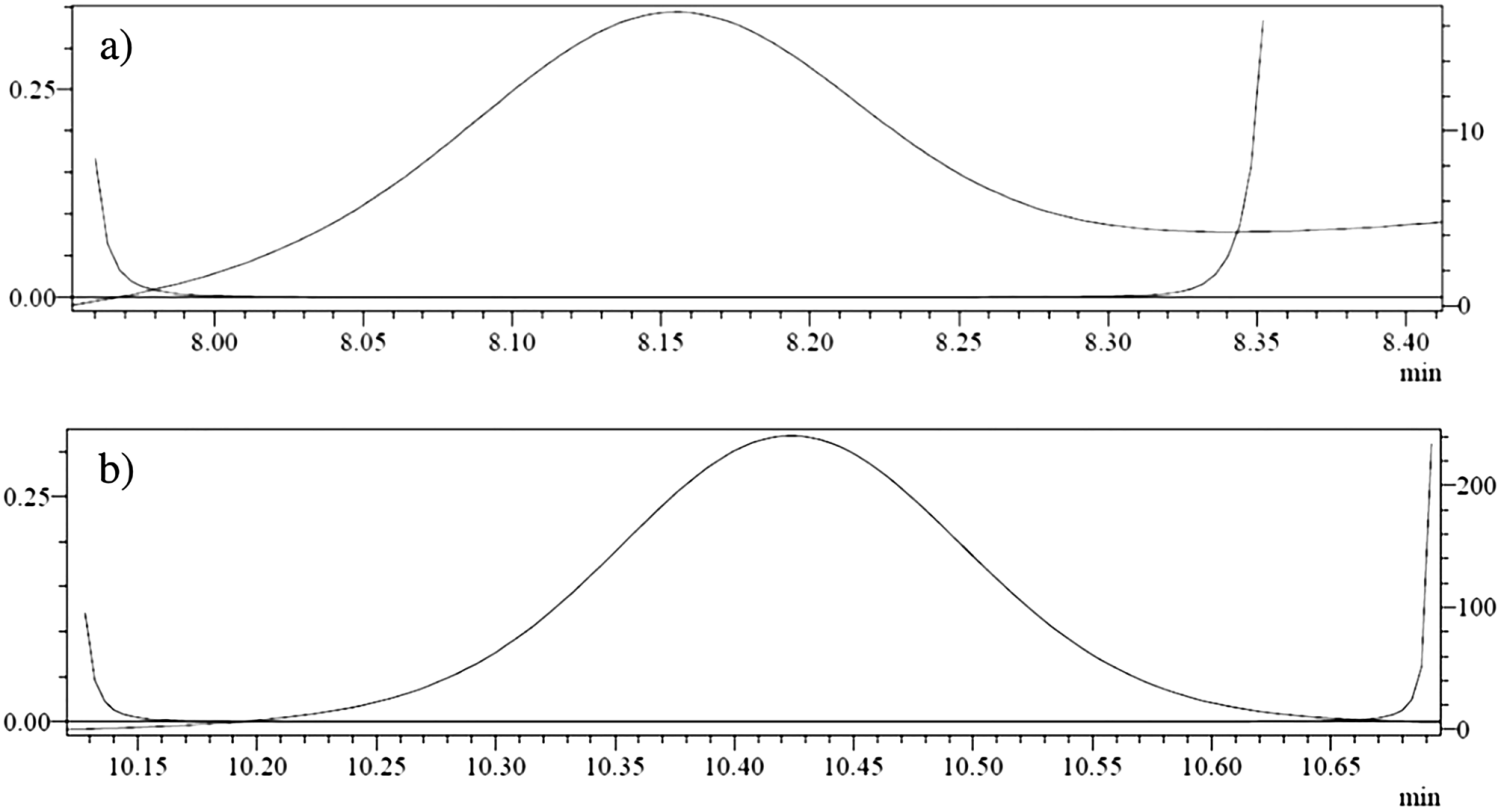
Spectral purity of the peak: (**a**) 6-thioguanine at 2000 ng/ml, (**b**) internal standard (peak purity mean 0.999—peak pure).

The spectrum of 6-TG and the internal standard taken in samples containing red blood cells are shown in Fig. 3, respectively. The purity of the peaks of the substance in the samples fortified with 6-TG and IS was analysed based on the compounds absorption spectrum of the tested and the internal standard. The results of this series of tests are shown in Fig. 4. The obtained maximum purity index (PPI, expressed in [%]) for 6-TG is 99.9% and for the internal standard, 99.9%. The PPI values (criterion: PPI > 95%) demonstrates the high specificity of the method.

In conclusion, the lack of peaks of substances that elute at and around the retention times of the test substances (6-TG) and the internal standard (6-BU) in the excipients proves the accurate specificity of the determination of both test compounds. The validity of this conclusion is confirmed by the data presented in the section on the verification of the method selectivity and the analysis of the ratio of signals obtained during the retention of analytes and the internal standard in the blank samples of various samples concerning those characterising the 6-TG fortified sample at the LLOQ (lower limit of quantification) level.

### Selectivity

**Figure 5:**
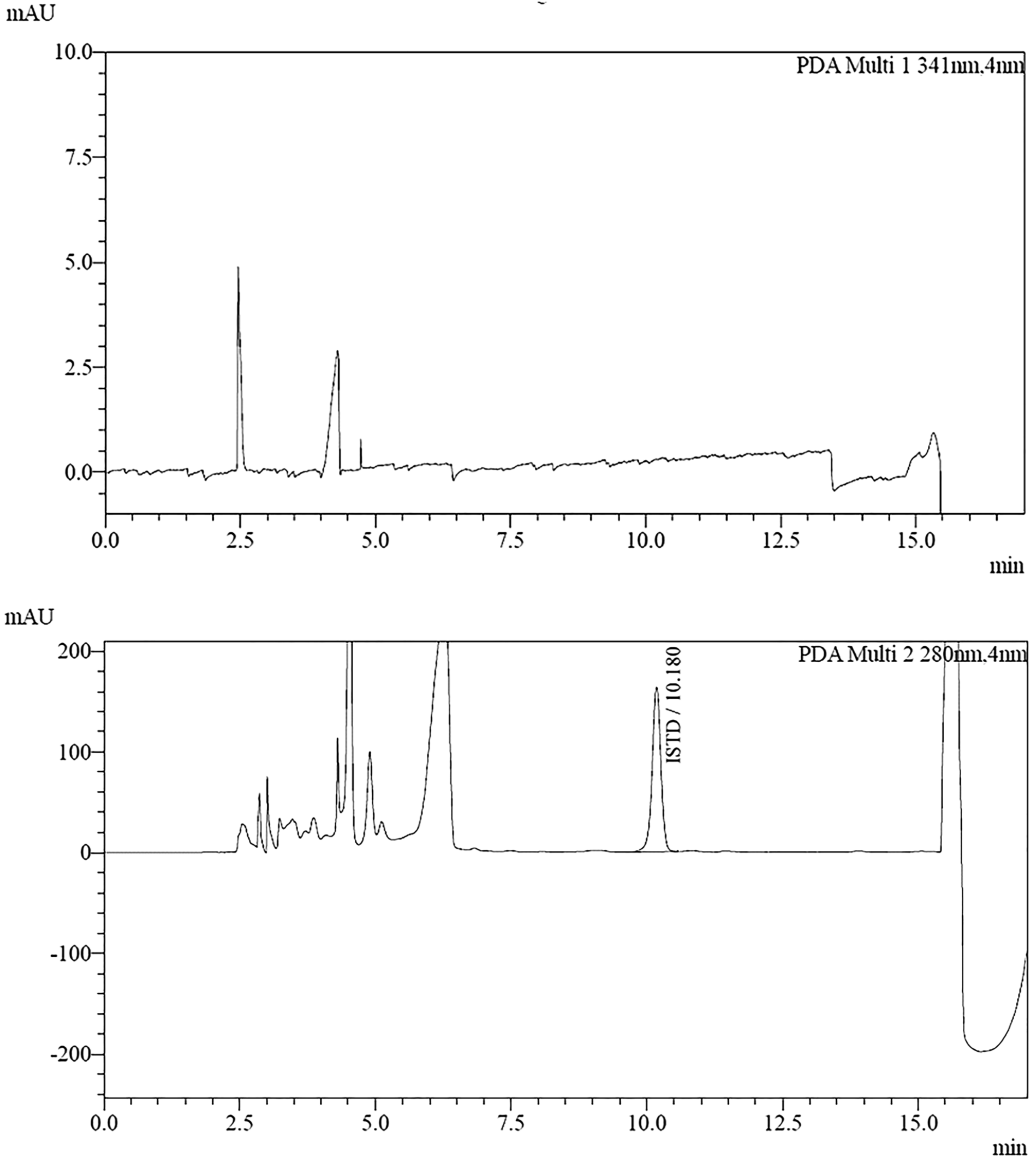
Chromatograms of a blank sample.

**Figure 6:**
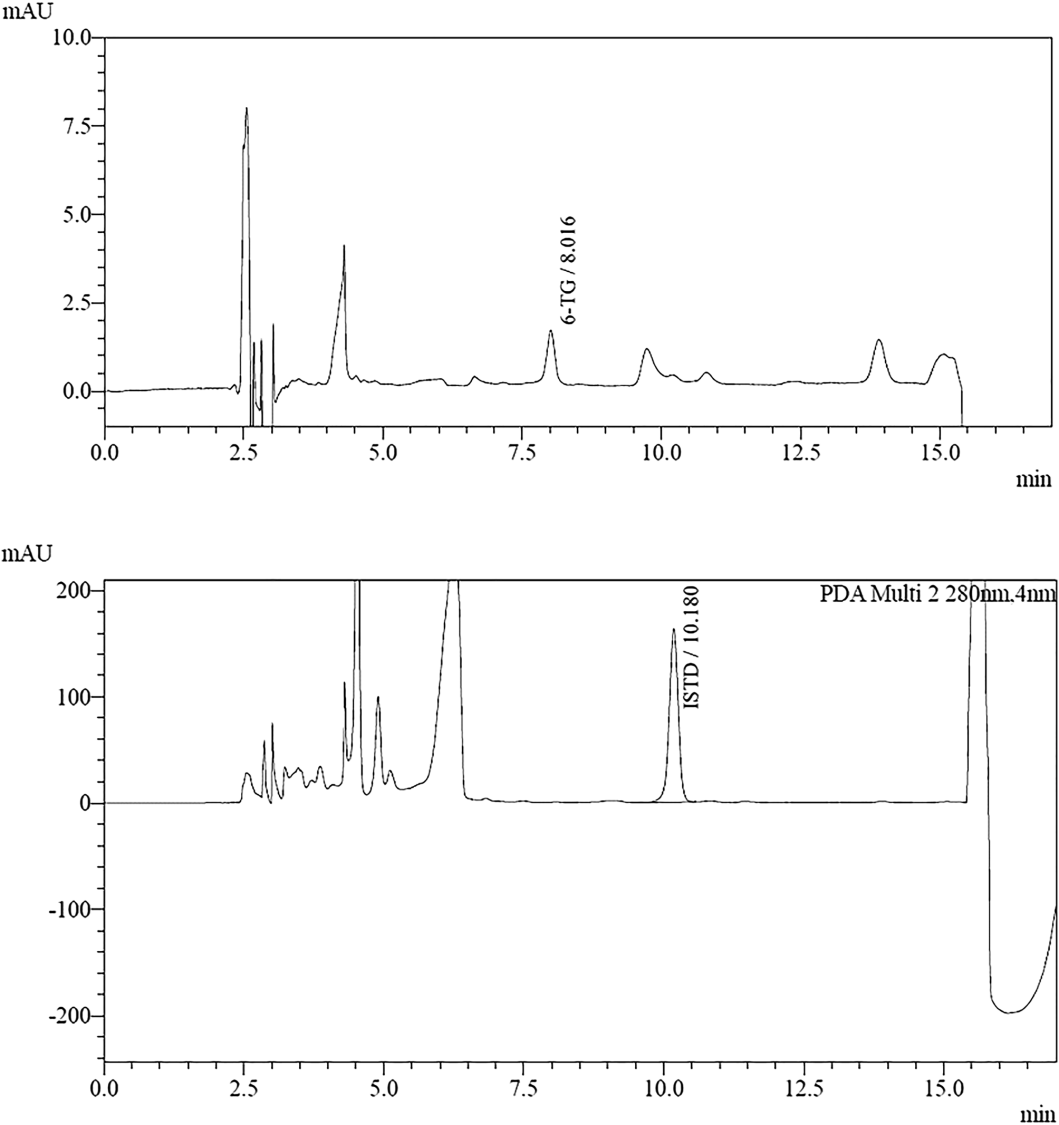
Chromatograms of erythrocyte samples fortified with 6-TG at the low level control.

According to the guidelines, the absence of interfering substances is determined by comparing the signals obtained during the analyte retention time and the internal standard for the blank sample and the sample at the LLOQ level (Figs. 5, 6). The detector response during analyte retention for the blank sample should be less than 20% of the signal found in the LLOQ sample and less than 5% of the signal during IS retention. In this case, it is 0% for 6-TG and 0% for IS, respectively. Thus, the validated procedure has good selectivity. Furthermore, analysis of the peaks of retention times of the interfering substances in all tested blank samples confirms a high degree of peaks of their separation from the peaks of both analytes and IS (resolution RS > 2). The acceptability criterion was met.

### Linearity range

Analysis of the linearity of the 6-thioguanine chromatographic method was carried out on blood enriched with test substances in the range that covers the therapeutic level of 6-TG concentration (100–450 pmol/8 × 10^8^ RBC). However, the levels of 6-thioguaninelevels vary widely,, and the range of the method has been extended and validated from 479 to 17,118 ng/mL (48 pmol/8 × 10^8^ RBC and 1650 pmol/8 × 10^8^ RBC, respectively). For this purpose, a series of working calibration solutions were analysed from the 6-TG standard at a concentration of 10 mg/mL with the addition of a precisely known and equal amount of the internal standard (IS standard in a solution of 100 µg/mL). Four calibration solutions were prepared independently for each concentration level and dosed onto the chromatographic system. To verify the precision of the calculation of the 6-TG concentration based on the equations of the calibration curves (calculated based on the average values of the 6-TG and internal standard surface areas), the results are summarised in Table 1. To facilitate the comparison, the “experimental” concentrations of the calibration solutions used for calibration are presented for chromatographic systems (C_6-TG_^EXP^ and C_6-TG_^CAL^). In addition, the tables contain concentration difference (SC), measurement bias (Bias), and CV.

**Table 1:** Comparison of experimental concentrations and those calculated using the 6-TG calibration curve equation.

Calibration solutions	C_6-TG_^EXP^ [ng/mL]	C_6-TG_^CAL^ [ng/mL]	Concentration difference (S_C_)	CV [%]	Bias [%]
1	479	401	78	4.90	− 16.3
2	822	901	− 79	9.11	9.1
3	2738	3014	− 276	4.67	10.1
4	4108	4260	− 152	1.37	3.7
5	10,808	9989	819	6.45	− 5.0
6	17,118	18,220	− 1102	2.68	6.4

From the data presented above, for calibration solutions 1 to 6, Bias is in the range of − 16.3 < Bias < 10.1 (for 6-TG). Considering that the criterion of acceptability of calibration solutions is to obtain a concentration calculated from the equation of the calibration curve not differing by more than 15% from the nominal concentration (in the case of the LLOQ level, the estimated concentration should not exceed 20% of the nominal concentration), the obtained results confirm the fulfilment of the criterion of acceptability for the tested compound.

### LOD and LOQ

**Figure 7:**
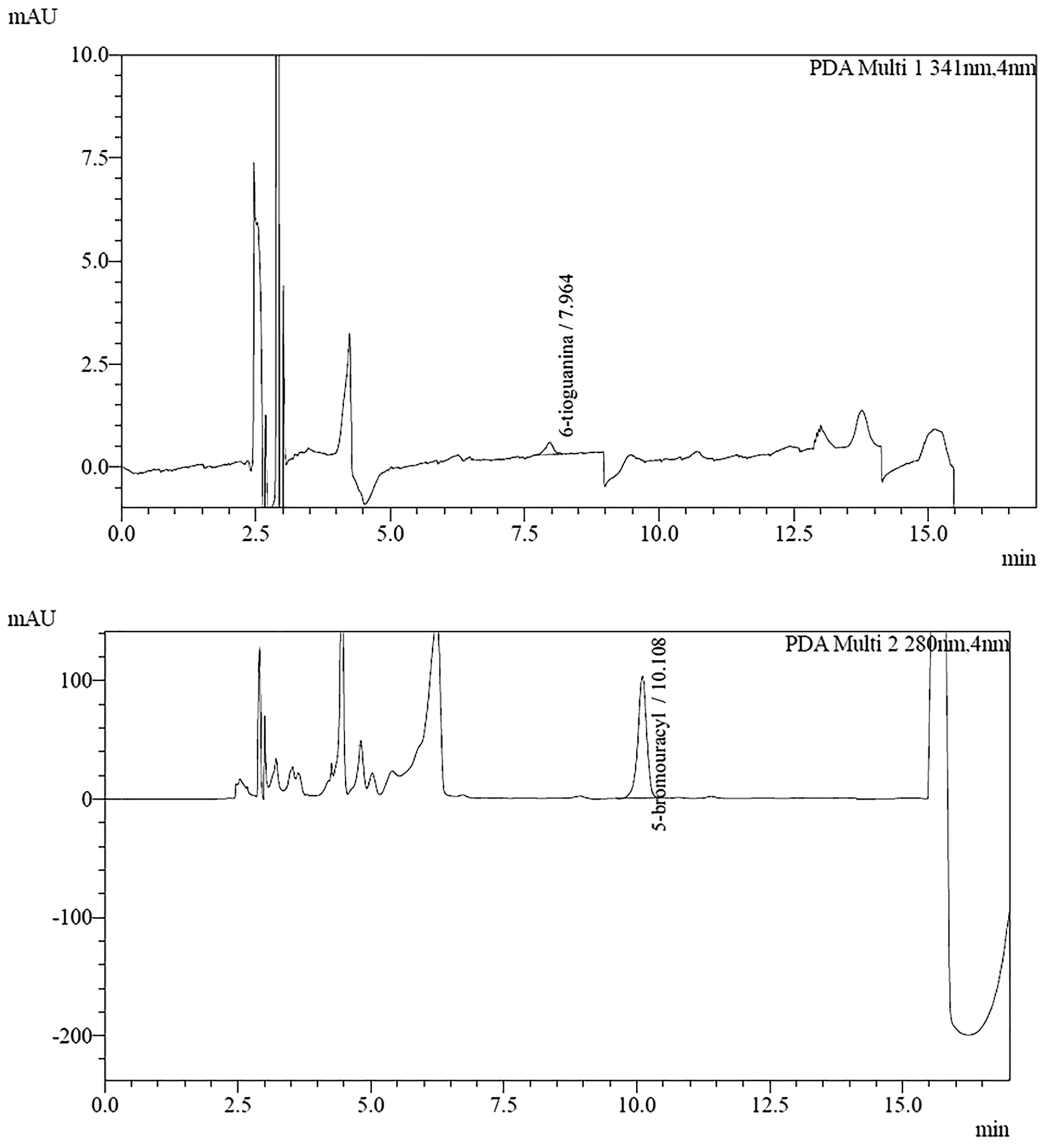
Chromatograms of the 6-TG (150 ng/mL) calibration solution (LOD) (7.6 min—6-TG; 10.0 min—IS).

**Figure 8:**
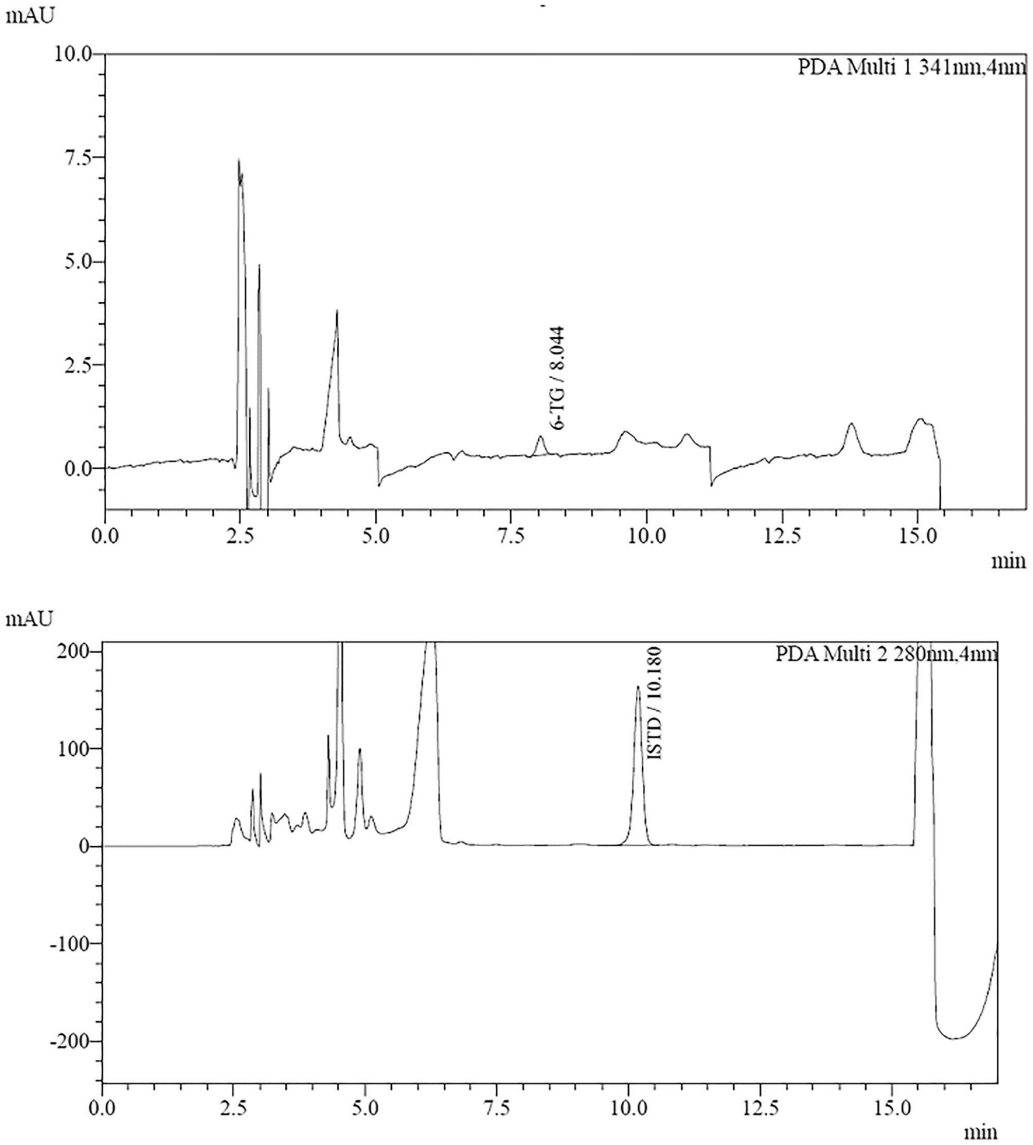
Chromatograms of the 6-TG (479 ng/mL) calibration solution (LOQ).

Based on the mathematical relationships mentioned in the Materials and Methods section, the LOD (Fig. 7) and LOQ (Fig. 8) values for 6-TG were 371 ng/mL and 1123 ng/mL, respectively. However, after obtaining the calculation results, it was checked that the EP s/n (signal-to-noise ratio) for the concentrations of 371 ng/ml and 1123 ng/ml, respectively, are much higher than the requirements assumed; it was decided to find a concentration that would meet these requirements. LOD of 150 ng/mL (equivalent to 14 pmol/8*10^8^ RBC) and EP s/n = 8 and LOQ of 479 ng/mL (equivalent to 46 pmol/8*10^8^ RBC) where EP s/n = 21. The results confirm the fulfilment of the acceptability criterion for the tested compound.

### Accuracy of the method (trueness)

The accuracy of the 6-TG analysis was evaluated by triplicate analysis of five different whole blood samples fortified with the test substance at three concentration levels covering the calibrated concentration range, i.e., 750 ng/mL; 4389 ng/mL, 12,540 ng/mL (equivalent to 73 pmol/8*10^8^ RBC; 423 pmol/8*10^8^ RBC, 1209 pmol/8*10^8^ RBC). The Bias for 6-TG is in the range of − 5.6 < Bias < 14.7. This means that the average value of the results did not differ from the nominal values by > 15%. There is no statistical difference between the mean concentrations of the results calculated for the concentration level and the known concentration value. The acceptability criterion for both compounds was met.

### Repeatability and intermediate precision

Based on the determined ratios of the 6-TG peak area to the internal standard and the equation of the calibration curves, concentrations of analytes were obtained, the mean values of which are summarised in Table 2, together with the statistical processing of the results obtained. The analysis of the results from the above tables shows that the repeatability of the 6-TG analysis at three levels of analyte concentrations (750 ng/mL, 4389 ng/mL and 12,450 ng/mL) expressed by the value of the coefficient of variation (CV) is in the range of 1.30–3.24%. Thus, the acceptance criterion is met.

**Table 2:** Repeatability of 6-TG analysis in a whole blood sample, free of a test substance, five times fortified with 6-TG at three concentration levels.

Sample no.	Precision of 6-TG analysis at the concentration level:		
	750 [ng/mL]	4300 [ng/mL]	12,450 [ng/mL]
1	816.86	3982.05	10,813.74
2	819.92	3846.12	10,758.41
3	852.15	3866.36	10,951.04
4	811.64	4331.29	12,195.43
5	782.45	4409.35	11,876.53
Repeatability (n = 5)	816.01 ± 2.82 [ng/mL]	4087.03 ± 2.29 [ng/mL]	11,319.03 ± 3.25 [ng/mL]
	CV = 3.08 [%]	CV = 1.30 [%]	CV = 3.24 [%]

The intermediate precision of the analysis of 6-TG in whole blood at three concentration levels expressed as a coefficient of variation (CV) is in the range of 4.19–5.78% for 6-TG. The research was carried out by different analysts using different standard working solutions, and the results are presented in Table 3. The values obtained for the coefficient of variation meet the acceptability criterion.

**Table 3:** Intermediate precision of 6-TG analysis in various fortified serum samples (*n* = 10) at three analyte concentration levels.

Series no.	Serum no.	Concentration level:		
		750 [ng/mL]	4300 [ng/mL]	12,450 [ng/mL]
1	1	816.86	3982.05	10,813.74
	2	819.92	3846.12	10,758.41
	3	852.15	3866.36	10,951.04
	4	811.64	4331.29	12,195.43
	5	782.45	4409.35	11,876.53
Mean		816.01	4087.03	11,319.03
CV [%]		3.08	1.3	3.24
2	1	729.94	4501.54	12,183.37
	2	725.87	4613.55	12,253.19
	3	726.17	4488.71	12,233.52
	4	736.33	4578.52	12,304.92
	5	727.29	4520.99	12,247.15
Mean		729.12	4540.66	12,244.43
CV [%]		0.44	0.98	0.24
Intermediate precision (n = 10)		772.6 ± 12.6 [ng/mL]	4313.9 ± 25.6 [ng/mL]	11,178.7 ± 0.039 [ng/mL]
		CV = 5.62 [%]	CV = 5.78 [%]	CV = 4.19 [%]

### Recovery

The surface areas of analytes and the internal standard obtained for five different EDTA blood samples spiked with 6-TG and IS at three concentration levels (750 ng/mL; 4389 ng/mL, 12,540 ng/mL) are presented along with the recoveries calculated for each level in Table 4.

**Table 4:** 6-TG/IS surface areas were obtained for blood samples fortified at three levels.

Blood sample no.	1	2	3	4	5
6-TG—level I (750 ng/mL)					
6-TG/IS surface area	0.00668	0.00616	0.00623	0.00659	0.00716
Recovery [%]	96.7	89.2	90.1	95.3	103.6
Average recovery [%]	95.0			CV [%]	6.1
6-TG—level II (4389 ng/mL)					
6-TG/IS surface area	0.04228	0.04368	0.04331	0.04503	0.04228
Recovery [%]	79.1	81.7	81	84.2	79.1
Average recovery [%]	81			CV [%]	2.6
6-TG—level III (12,540 ng/mL)					
6-TG/IS surface area	0.16352	0.16731	0.15545	0.15546	0.15676
Recovery [%]	95.2	97.4	90.5	90.5	91.2
Average recovery [%]	92.9			CV [%]	3.4

The comparison of the average values of 6-TG and IS recovery obtained at individual concentration levels of both analytes shows that for all compounds, it is comparable and independent of the concentration of the analyte. The average recovery of 6-TG calculated for three concentration levels of this compound is 79.1–103.6%, respectively. The acceptability criterion was fulfilled.

### Carryover

The signals read during analyte retention time and IS in the blank sample dosed after the calibration solution at the highest concentration level were collected. The analysis of the results revealed the lack of peaks in the chromatogram corresponding to the blank sample. The calibration solution with the highest analyte concentration was dosed immediately after dosing. It proves the absence of sample transfer, which confirms that the acceptance criterion is fulfilled.

### Stability

Stock solutions have been stable for 30 days. Standard to calibration and control—whole blood samples spiked with 6-TG at low (650 ng/mL) and high (8906 ng/mL) IS levels were used for stability checks and stored for 24 h in an autosampler at 5 °C. The choice of using concentrations of 650 ng/mL and 8906 ng/mL for stability evaluation was based on several considerations. First, these concentrations fall within the linear range of the analytical method used for the evaluation. It is essential to assess stability at concentrations within this range to ensure accurate and reliable results during routine analysis. Second, the decision to evaluate the stability at these specific concentrations was influenced by the observation of such concentrations in samples from several patients. In clinical practice, it is not uncommon to encounter a diverse range of analyte concentrations in patient samples. Therefore, evaluating the stability at concentrations commonly found in real-world samples provides valuable information on the performance of the assay under realistic conditions. The detector response obtained for the 6-TG and IS peaks was compared in the sample exposed to the verified parameter. Recovery [%] was adopted as the acceptance criterion for the tested solutions about the freshly prepared solution in the range indicated above in the recovery tests. The results are presented in Table 5 as the value of the independently prepared samples. The analysis of the data presented shows that: (a) samples of the internal standard, as well as samples of basic and working standards, should not be frozen; precipitated substances were observed in the tested samples, which did not dissolve after leaving at room temperature, as well as after using vortex; and (b) the whole blood sample fortified with low and high level EDTA is very stable. Stored in an autosampler feeder (5 °C), it is stable for up to 24 h. In conclusion, whole blood samples fortified at two concentration levels of 6-TG are stable for up to 24 h at 5 °C.Patient samples were tested for 10 days, performing daily tests (samples were stored at room temperature, in the refrigerator, and frozen). Analysis revealed that samples could not be frozen (recovery below 50%). Samples stored at room temperature for 2 days recovered above 50% (for samples stored for more than 2 days). Samples stored for 7 days in the refrigerator showed appropriate recovery. The stability of the research standards (30 days—freezer) and the stability of the prepared test samples for analysis within 24 h were assessed. The study allowed for determining the stability of the tested sample (7 days at room temperature).

**Table 5:** The stability of the whole blood sample in the autosampler feeder (temp. 5 °C) was fortified at 650 ng/mL and 8906 ng/mL concentrations of 6-TG.

Time [h]	Concentration level of 6-TG: 650 ng/mL			Concentration level of 6-TG: 8906 ng/mL		
	P6-TG [mAU*min]	PIS [mAU*min]	C6-TG [ng/mL]	P6-TG [mAU*min]	PIS [mAU*min]	C6-TG [ng/mL]
0	17,482	2,597,300	650	188,753	1,838,724	8906
4	16,820	2,587,300	651	188,763	1,838,722	8907
Recovery [%]	96.2	99.6	100.2	100.0	100.0	100.0
8	17,520	2,579,850	640	188,654	1,800,522	8806
Recovery [%]	100.2	99.3	98.5	99.9	97.9	98.9
24	17,456	2,678,000	642	186,584	1,845,652	8807
Recovery [%]	99.9	103.1	98.8	98.9	100.4	98.9
48	17,052	2,687,001	630	175,684	1,820,646	8654
Recovery [%]	97.5	103.5	96.9	93.1	99.0	97.2

Before developing the method described in this paper for the rapid and sensitive determination of 6-TG in red blood cells, we tested many research protocols in scientific articles on sample preparation, knowledge of sample stability, and analytical methods. Modified, validated methods from all these articles were used, adapting, and extending our approach. These studies inspired us and gave us a broad picture of method optimization.

The developed analytical method is faster compared to that contained in the work by Cangemi et al.^31^ The total time of the test takes about 40 min and allows the use of a smaller volume of sample injected into the chromatographic column, which in turn can extend the life of the column, resulting in savings during routine maintenance.

Compared to the publication by Franzin et al.^32^, our method allows for a broader range of tested samples, lower limits of detection (LOD) and quantification (LOQ), as well as the ability to determine 6-TG with a resolution greater than 2.0.

Stefan et al.^33^ focused on different aspects of sample stability and reference range in various study groups; our approach minimized the number of chemicals used in test samples and prompted efforts to reduce sample volumes. This was an important goal, especially for samples from children who received appropriate medications. Assuming the use of 200 µL in our study, it is possible to take a tiny sample from small patients.

Today, where the possibility of simple and relatively quick analysis plays an important role, especially when drug concentrations in a large group of patients, it is crucial that validation work is underway to further reduce the required sample volume.

## Conclusions

The research aimed to develop and validate a fast and sensitive HPLC-based methodology to determine 6-TG in red blood cells. The lower limit of quantification (LLOQ) was 450 ng/mL, and the upper limit of quantification (ULOQ) was 17,000 ng/mL 6-TG in whole blood. The calculated LOD and LOQ values were 150 and 479 ng/mL of whole blood, respectively. The calibration curve was linear (R > 0.99) over the 6-TG concentration range of 479–17,118 ng/mL in whole blood. The intra-day and inter-day precision were within generally accepted criteria for a bioanalytical method (< 15%).

The methodology helps assess the quality of therapy by determining the concentration of 6-thioguanine. The method considers the level of red blood cells (RBC) in each patient in the final result of the test sample. The levels of metabolites can vary significantly. The results note that sometimes low concentrations of 6-thioguanine are maintained for a long time during treatment with this drug. The method allows for the determination of a wide range of concentrations, resulting in great diversity and remarkable possibilities of this method.

The developed method is simple, fast, effective, and reliable. It is suitable for analyzing this antileukemia drug over a wide therapeutic concentration range in whole blood. Its usefulness has been proven by applying it to the analysis of actual blood samples fortified with 6-TG. Therefore, the proposed method is suitable for determining 6-TG in whole blood for drug level monitoring and pharmacokinetic studies.

## Materials and methods

### Chemical and reagents

5-bromouracil (5-BU), used as an internal standard (IS), was supplied by Sigma-Aldrich (LOT 852473, 98% purity). 6-thioguanine (6-TG), as a stock solution, was supplied from LGC Standards (LOT MM0865.0, 99% purity). Acetonitrile (ACN) and orthophosphoric acid 85%, both HPLC grade, were purchased from VWR (LOT 83640 and 15315). Perchloric acid (70%) was purchased from Merck (LOT 30755-M). Deionized water from Milli-Q (Millipore) was used (LOT 83645). Red blood cells (RBCs) were used to validate the method. Whole blood samples taken from patients administered 6-thioguanine were studied within the scope of routine drug monitoring examinations.

### Standard drug solutions

A stock solution of 6-TG (C_p 6-TG)_ at a concentration of 6 mg/mL in 0.1 M NaOH. To prepare a working solution of 6-TG (C_r 6-TG_), appropriate dilutions were made in the linearity range of 50 ng/mL to 2000 ng/mL. Water was used as the dilution solvent. An internal standard of 5-bromouracil (C_r IS_) at 1 mg/mL concentration in 5 mL of 0.1 M NaOH. Standards and internal standards were stored at 2–8 °C.

### Red blood cells isolation

Whole blood was collected from healthy volunteers and collected in tubes containing sodium heparin, cubital or cephalic veins were preferred for blood sampling. For 1 ml of whole blood, 1 ml of saline was added and then centrifuged at 5000 rpm for 5–10 min at 4 °C; the resulting plasma was discarded. Particular attention was paid not to disturb the layer of white blood cells above the erythrocytes. Centrifugation was repeated twice to remove the plasma and leave only red blood cells.

### Preparation of calibration and control samples

A series of working calibration solutions were obtained from the 6-thioguanine standard at a concentration of 0.05 mg/ml (C_r 6-TG_) with the addition of a precisely known and equal amount of the internal standard (IS standard concentration in a solution of 1 mg/ml) and red blood cells without study substances and internal standard. The red cells added to the calibration serve as a template to better replicate the conditions of blood samples. This affects the solubility and interaction of the analysis with other blood components, which is essential for calibrating the analytical method. In this case, red blood cells were added as a matrix element to adapt the samples to more realistic laboratory conditions. The volume ratios used to prepare the working calibration solutions are summarised in Table 6.

**Table 6:** Volume ratios are used to prepare working calibration solutions.

Calibration solutions	Whole blood volume [µl]	Volume of C_r 6-TG_ solution [µl]	Volume of IS solution [µl]	Volume of saline [ul]	Volume of DTT [µl]	Volume of HClO4 [µl]	Total vol. samples [µl]	6-TG concentration in whole blood [ng/ml]
1	200	0	10	140	25	50	425	0
2	200	40	10	100	25	50	425	150
3	200	40	10	100	25	50	425	479
4	200	40	10	100	25	50	425	822
5	200	40	10	100	25	50	425	2738
6	200	40	10	100	25	50	425	4108
7	200	40	10	100	25	50	425	10,808
8	200	40	10	100	25	50	425	17,118

Control samples were prepared in whole blood containing neither study substances nor internal standard by fortification with 6-TG standard solutions at a concentration of 0.05 ng/mL (C_r_ 6-TG) with the addition of a precisely known and equal amount of the internal standard concentration (concentration of the IS standard in a solution of 1 mg/mL). The volume ratios used to prepare the control samples are summarised in Table 7.

**Table 7:** Volume ratios were used to prepare control samples.

Control	Whole blood volume [µl]	Volume of C_r 6-TG_ solution [µl]	Volume of IS solution [µl]	Volume of saline [ul]	Volume of DTT [µl]	Volume of HClO4 [µl]	Total vol. samples [µl]	6-TG concentration in whole blood [ng/ml]
1	200	40	10	100	25	50	425	750
2	200	40	10	100	25	50	425	4300
3	200	40	10	100	25	50	425	12,450

### Sample preparation procedure for chromatographic analysis

Pipette exactly 200 µl of whole blood, 140 µl of saline (for calibration and control samples, add the appropriate amount of working solution and a correspondingly smaller amount of saline), 10 µl of internal standard solution (C_r_ IS), 25 µl of DTT (ditriethiol—1.1 M solution) and 50 µl of perchloric acid (65%) in an eppendorf tube. The tube contents were thoroughly mixed using a vortex (30 s) and centrifuged (12,500 rpm, 10 min). The supernatant was then transferred to Eppendorf and heated at 100 °C for 40 min. After this time, the sample was cooled and transferred to the HPLC plate. The vial contents were thoroughly mixed using a shaker (10 s) and then placed in a vial of the autosampler feeder. Essential to ensure that the liquid in the insert does not contain air bubbles.

### Chromatographic conditions

All analyses were performed on the Nexera X2 liquid chromatographic system model (Shimadzu, Japan), equipped with a SIL-30ACXR autosampler, LC-30 AD pump, and DGU- 20A5R degasser, a model CTO-20AC oven, and an SPD30A model photodiode array (PDA) detector. Data were acquired and processed with LabSolutions software.

Separations were carried out using a Luna™, Phenomenex analytical column (250 × 4.6 mm, 5 μm) analytical column. A mobile phase consisting of 6.8 g of ACN/buffer KH2PO4 was used in 1L 3/97% v/v with the addition of 50ul H3PO4 at a constant flow rate of 1.0 mL/min. The stationary phase was acetonitrile. The column was kept at wavelengths of 30 °C, and the eluents were monitored at 280 nm (5-BU analysis) and 341 nm (6-TG analysis). The injection volume was 50 μl.

### Assay validation

Method validation was carried out under the guidelines EMA^34^, ICH^35^ and ISO 17025^36^ in selectivity/specificity, linearity range, limits of detection and quantification, accuracy, precision, extraction recovery, carryover, and stability.

### Selectivity/specificity

A particular/specific analytical method should allow the resolution and detection of the drug of interest and the internal standard in the occurrence of potential coadministered drugs and coeluting endogenous compounds in the matrix^37^.

The specificity of the determination of the 6-thioguanine method was evaluated by analysing serum samples without test substances and internal standard (negative test) and the resolution between the peaks of aqueous solutions of test substance standards (positive test) and aqueous solutions of the internal standard (positive test), as well as between these peaks and whole blood samples with the addition of test substances (samples fortified with 6-thioguanine) and serum samples fortified with an internal standard.

Five blank samples from different patients were analysed to verify the method's selectivity. In each chromatogram, the signals obtained during the retention time of 6-TG and IS were integrated and compared with those obtained in the sample enriched with the level of test substance at the LLOQ (lower limit of quantification).

### Linearity range

The linearity of an analytical method means the direct proportional response of the results obtained to the analyte concentrations in an appropriate calibration set. The linearity study of the 6-thioguanine chromatographic method was carried out on blood enriched with test substances in the range covering the therapeutic level of 6-TG concentration (100–450 pmol/8 × 10^8^ RBC), according to the CLSI guidelines^38^. However, 6-thioguanine levels varied significantly, and the range of the method was extended and validated from 479 to 17,118 ng/mL (48 pmol/8 × 10^8^ RBC and 1650 pmol/8 × 10^8^ RBC, respectively). In converting the concentration range in pmol/8 × 10^8^ RBC to ng/mL, the molar mass of 6-thioguanine should be considered. Convert pmol to ng: divide the concentration range in pmol/8 × 108 RBC by the number of red blood cells (8 × 10^8^) to get the concentration in pmol/RBC. The concentration in pmol/RBC was then multiplied by the molar mass of 6-thioguanine to convert to ng/RBC. Convert ng/RBC to ng/mL: concentration in ng/RBC was divided by the blood volume (mL) used for the analysis, and the results were adjusted to the patient’s red blood cell (RBC) count.

For this purpose, a series of working calibration solutions obtained from the 6-TG standard at a concentration of 10 mg/mL with the addition of a precisely known and equal amount of an internal standard (concentration of the IS standard in a solution of 100 µg/mL) was analysed. Four calibration solutions were independently prepared for each concentration level and dosed onto the chromatographic system. The minimal range should cover analyte concentrations from 50 (LLOQ, lower limit of quantitation) to 150% (ULOQ, upper limit of quantification).

The limits of detection (LOD) were determined based on the following mathematical relationship: LOD = (3.3 × SD)/*a*. On the other hand, for quantification (LOQ), it looked as follows: LOQ = (10 × SD)/*a*. SD stands for the standard deviation of the regression line, and *a* is the directional coefficient of a calibration curve.

### Accuracy of the method (trueness)

The accuracy of an analytical method (Bias) is the closeness of the average results obtained by a given method to the actual concentration of the analyte in the sample. It is determined based on analyses of samples containing precisely known concentrations of the analyte and analyses of the so-called control samples. Factors affecting the accuracy of a given method are the components of both systematic and random errors. Trueness, the degree of agreement between the traditional value and the experimental mean, is measured as a percentage deviation from the accepted reference value (Bias)^37^. The student’s t-test was used for statistical evaluation. The acceptance criterion for biological samples: the mean value of the results should not deviate from the nominal value by more than 15% (20% for LLOQ).

### Precision

Precision is the degree of dispersion between measurements of a series of multiple samples of the same homogeneous sample under the same experimental conditions. Usually expressed as inaccuracy and reported as a relative standard deviation (RSD, CV). Precision can be repeatability or intermediate precision. The paper defines repeatability and intermediate precision.

Repeatability means accuracy under specified operating conditions over a short period (usually within one day of analysis). To determine the method's reproducibility, samples of whole blood fortified with various amounts of 6-TG containing precisely known concentrations of both compounds and the internal standard were used. For this purpose, five control samples of whole blood not containing the test substance were fortified with 6-TG at three concentration levels (750 ng/mL, 4389 ng/mL and 12,450 ng/mL) and subjected to triple chromatographic analysis.

Intermediate precision studies were performed for 2 days on 5 different samples of whole blood, which, as before, were enriched at three concentration levels, including low, medium and high levels of 6-TG in whole blood, and were subjected to triple chromatographic analysis. The analyses were performed by different analysts using different standard solutions. The intermediate precision of the analysis of 6-TG in whole blood at three concentration levels is expressed by the coefficient of variation (CV).

### Extraction recovery

The extraction recovery was determined as the percentage of the analyte reaction after sample preparation compared to the response of the analyte present in the standard solution at a known concentration. Extraction recovery of 6-TG extraction from serum samples was determined at three levels of analyte concentration, 750 ng/mL, 4389 ng/mL and 12,450 ng/mL, relative to an aqueous solution of a mixture of 6-TG and IS.

### Carryover

Carryover refers to the contamination of the sample being analysed with a substance present in the autosampler loop after the previous analysis. These tests are carried out using two alternating dosing solutions. The first is the calibration solution with the highest concentration of the test substance, and the second is a blank sample containing neither the test substance nor the internal standard. The absence of peaks in the chromatogram of the blank sample indicates a lack of transfer.

### Stability

Stability means the chemical stability of the analyte in each matrix under certain conditions for particular time intervals^37^. The stability of whole blood samples fortified at 650 ng/mL and 8906 ng/mL concentration levels of 6-TG with the internal standard (IS) was tested, adequately prepared and stored for 24 h in the autosampler at 5 °C. The stability analysis compared the detector response obtained for the 6-TG and IS peaks in the sample exposed to the verified parameter. The acceptance criterion was the recovery determined for the tested solutions about the freshly prepared solution in the range indicated above in the recovery tests.

### Ethical approval and consent to participate

This study followed the Polish Council of Medical Research Agencies guidelines. We involve human volunteers by the guidelines of the Declaration of Helsinki. All the participants were aware of the research purpose. Informed consent was obtained from all participants/or participants’ legal guardians. The methodology has been developed for commercial analyses performed in a certified diagnostic laboratory. Neither the Polish Centre of Accreditation (PN-EN ISO 15189) nor the National Chamber of Laboratory Diagnosticians does not indicate the need to obtain the consent of the bioethics committee. Generally, blood and its fractions and urine are the most common material for routine determinations in this type of laboratory unit. Polish legislation (Act on clinical trials of medicinal products for human use 13/01/2023) states that ethics approval is not required in this case.

## Data Availability

The datasets used and/or analyzed during the current study are available from the corresponding authors on reasonable request.
